# Supplementary material for: An Influenza A virus can evolve to use human ANP32E through altering polymerase dimerization
Source: Nat Commun. 2023 Oct 10;14:6135. doi: 10.1038/s41467-023-41308-4 (PMC10564888; doi:10.1038/s41467-023-41308-4)

Supplementary Information

Supplemental Figure 1: Viral titres during experimental evolution.

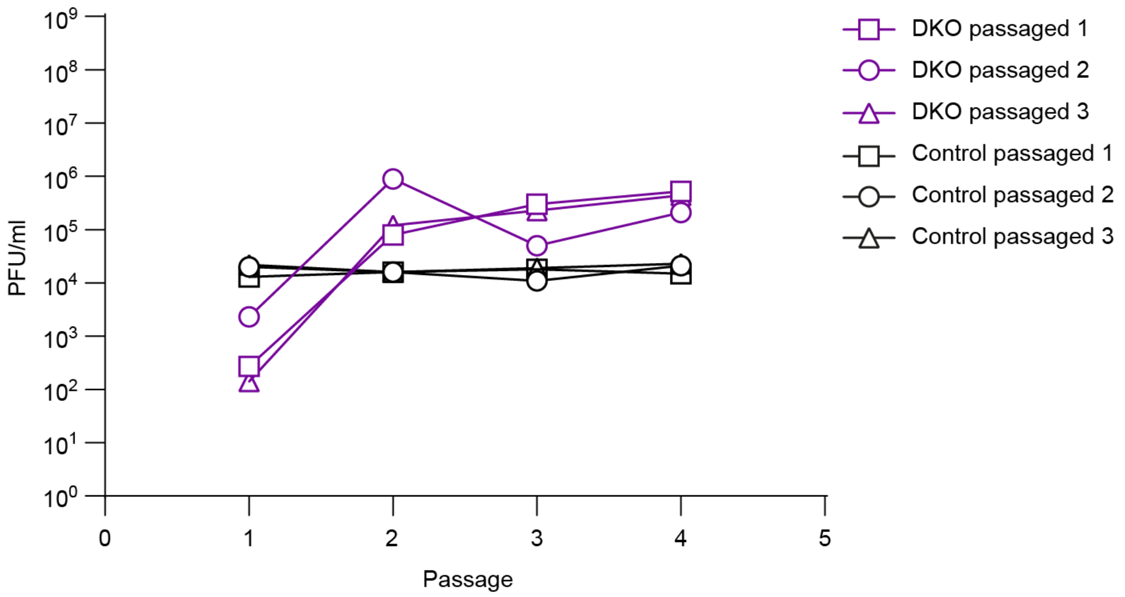

Three populations of Tky05 viruses were passaged on DKO and control cells respectively. Following each 72-hour passage, each population was grown on MDCK cells and the titre calculated, n=1 well. The titre was used to calculate the appropriate dilution for each population for subsequent passages. Source data are provided as a Source Data file.

Supplemental Figure 2: Increased WT PB1 concentration does not increase polymerase activity in DKO cells.

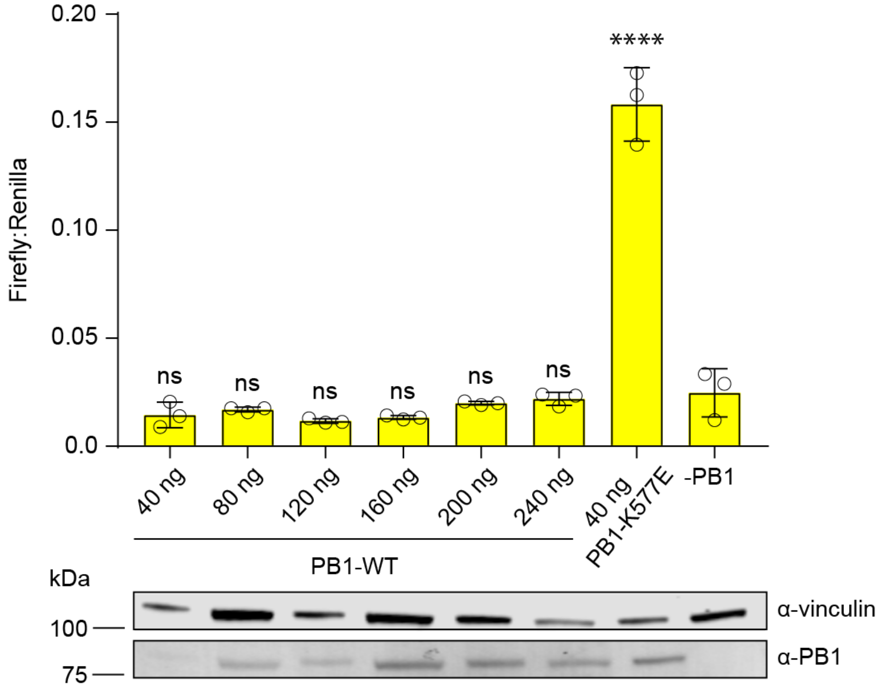

Minigenome assay performed in 24-well plates of eHAP DKO cells transfected with pCAGGS Tky05 PB2 (0.04 µg), PB1 WT (0.04 µg to 0.24 µg) or K577E (0.04 µg), PA (0.02 µg), NP (0.08 µg), reporter pPolI-luc (0.08 µg) and control pCAGGS-*Renilla* luciferase (0.04 µg). Data presented is representative of n=3 wells, statistical significance was determined by multiple comparisons of a one-way ANOVA, * P<0.05, ** P<0.01, *** P<0.001, ****P<0.0001. Western blot showing expression of vinculin and PB1 from matched experiment conducted in DKO cells. Source data are provided as a Source Data file.

Supplemental Figure 3: Partial sequence alignment of human, chicken and mouse ANP32 proteins.

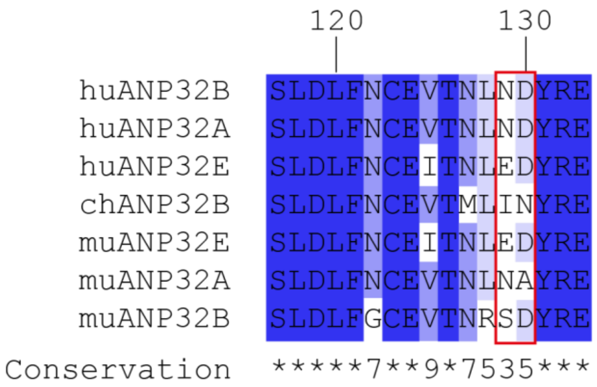

Sequence alignment comparing species variants of ANP32A, ANP32B and ANP32E proteins. Blue shading is representative of % identity. Generated using Clustal Omega and Jalview. Source data are provided as a Source Data file.

Supplemental Figure 4: Gluc1 and Gluc2 fusions reduce polymerase activity similarly for WT and K577E polymerases.

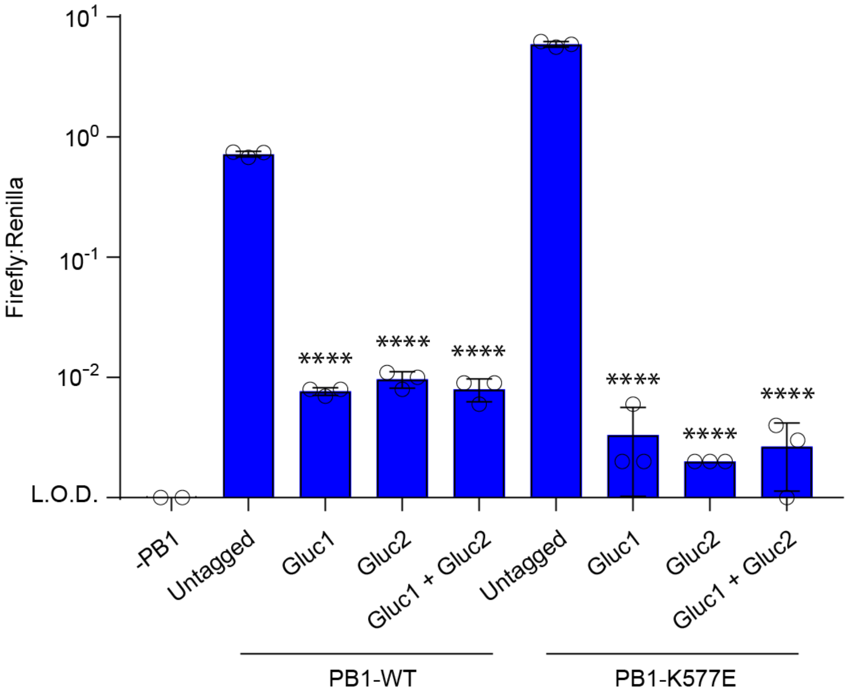

Minigenome assays comparing the effect of the Gluc1 and Gluc2 fusions on Tky05-WT and Tky05-PB1-K577E polymerase activity. Assays were conducted in TKO cells transfected with pCAGGS Tky05 PB2 (0.04 µg), PB1 WT/PB1 WT-Gluc (0.04 µg to 0.24 µg) or K577E/K577E-Gluc (0.04 µg), PA (0.02 µg), NP (0.08 µg), reporter pPolI-luc (0.08 µg) and control pCAGGS-*Renilla* luciferase (0.04 µg) supplemented with 0.04 µg huANP32B-FLAG. Data presented is representative of n=3 wells, statistical significance was determined by multiple comparisons of a one-way ANOVA comparing PB1-WT to PB1-K577E, * $P<0.05$, ** $P<0.01$, *** $P<0.001$, ****$P<0.0001$. Source data are provided as a Source Data file.

Raw images

Supplemental Figure 2

anti-PB1

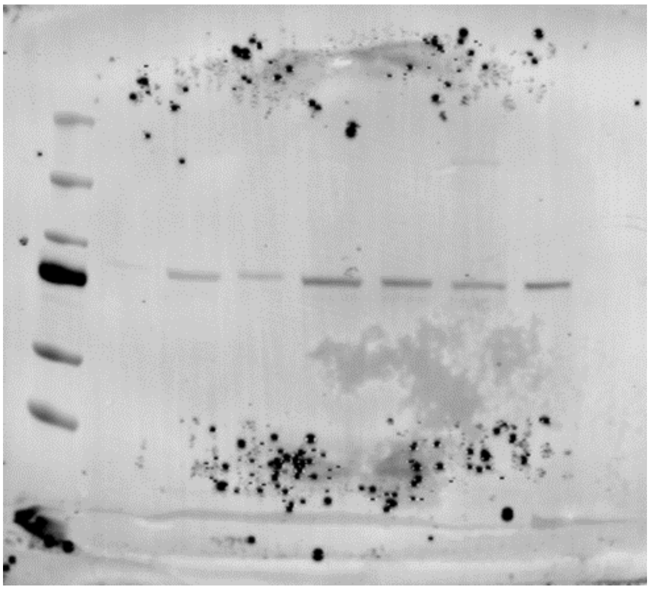

anti-vinculin

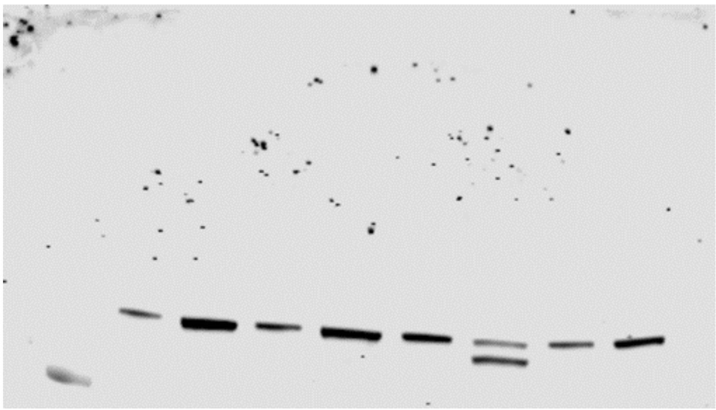

Supplement: Supplementary file 1 — Supplementary Information [file 41467_2023_41308_MOESM1_ESM.pdf]
