# Supplementary material for: An Influenza A virus can evolve to use human ANP32E through altering polymerase dimerization
Source: Nat Commun. 2023 Oct 10;14:6135. doi: 10.1038/s41467-023-41308-4 (PMC10564888; doi:10.1038/s41467-023-41308-4)
Supplement: Supplementary file 3 — Reporting Summary [file 41467_2023_41308_MOESM3_ESM.pdf]

Corresponding author(s): Carol Sheppard
Last updated by author(s): Jun 5, 2023

Reporting Summary

Nature Portfolio wishes to improve the reproducibility of the work that we publish. This form provides structure for consistency and transparency in reporting. For further information on Nature Portfolio policies, see our Editorial Policies and the Editorial Policy Checklist.

Statistics

For all statistical analyses, confirm that the following items are present in the figure legend, table legend, main text, or Methods section.

n/a	Confirmed	
☐	☒	The exact sample size (*n*) for each experimental group/condition, given as a discrete number and unit of measurement
☐	☒	A statement on whether measurements were taken from distinct samples or whether the same sample was measured repeatedly
☐	☒	The statistical test(s) used AND whether they are one- or two-sided<br>*Only common tests should be described solely by name; describe more complex techniques in the Methods section.*
☒	☐	A description of all covariates tested
☐	☒	A description of any assumptions or corrections, such as tests of normality and adjustment for multiple comparisons
☐	☒	A full description of the statistical parameters including central tendency (e.g. means) or other basic estimates (e.g. regression coefficient) AND variation (e.g. standard deviation) or associated estimates of uncertainty (e.g. confidence intervals)
☐	☒	For null hypothesis testing, the test statistic (e.g. *F*, *t*, *r*) with confidence intervals, effect sizes, degrees of freedom and *P* value noted<br>*Give P values as exact values whenever suitable.*
☒	☐	For Bayesian analysis, information on the choice of priors and Markov chain Monte Carlo settings
☒	☐	For hierarchical and complex designs, identification of the appropriate level for tests and full reporting of outcomes
☒	☐	Estimates of effect sizes (e.g. Cohen's *d*, Pearson's *r*), indicating how they were calculated

Our web collection on statistics for biologists contains articles on many of the points above.

Software and code

Policy information about availability of computer code

Data collection	Geneious 11.1.5, MARS data collection/analysis software, LiCor Empiria Studio 2.2
Data analysis	Prism v7, Clustal Omega, Jalview 2.10.5, ChimeraX

For manuscripts utilizing custom algorithms or software that are central to the research but not yet described in published literature, software must be made available to editors and reviewers. We strongly encourage code deposition in a community repository (e.g. GitHub). See the Nature Portfolio guidelines for submitting code & software for further information.

Data

Policy information about availability of data

All manuscripts must include a data availability statement. This statement should provide the following information, where applicable:
- Accession codes, unique identifiers, or web links for publicly available datasets
- A description of any restrictions on data availability
- For clinical datasets or third party data, please ensure that the statement adheres to our policy

GenBank accession numbers OR079445-OR079485
PDB: 6XZP https://doi.org/10.2210/pdb6XZP/pdb
PDB: 6QX8 https://doi.org/10.2210/pdb6QX8/pdb

Research involving human participants, their data, or biological material

Policy information about studies with human participants or human data. See also policy information about sex, gender (identity/presentation), and sexual orientation and race, ethnicity and racism.

Reporting on sex and gender	N/A
Reporting on race, ethnicity, or other socially relevant groupings	N/A
Population characteristics	N/A
Recruitment	N/A
Ethics oversight	N/A

Note that full information on the approval of the study protocol must also be provided in the manuscript.

Field-specific reporting

Please select the one below that is the best fit for your research. If you are not sure, read the appropriate sections before making your selection.

☒ Life sciences ☐ Behavioural & social sciences ☐ Ecological, evolutionary & environmental sciences

For a reference copy of the document with all sections, see nature.com/documents/nr-reporting-summary-flat.pdf

Life sciences study design

All studies must disclose on these points even when the disclosure is negative.

Sample size	No statistical method was used to predetermine sample size for in vitro experiments.<br>For animal experiments, a resource equation was used to determine group size for weight loss output, and a power calculation using G Power was used to determine groups size for measuring mean virus titres in lungs.
Data exclusions	No data were excluded from the analyses.
Replication	All mini-genome assays, split-luciferase assays, co-immunoprecipitation assays were conducted with triplicate wells and repeated a minimum of 3 times.<br>All experiments using live virus were conducted with triplicate wells and repeated a minimum of two times.<br>qRT-PCR experiments were conducted with triplicate wells with 3 biological repeats.<br>Animal experiments were conducted once using group sizes sufficient for adequate power to detect differences.
Randomization	The experiments were not randomized.
Blinding	The Investigators were not blinded to allocation during experiments and outcome assessment.

Reporting for specific materials, systems and methods

We require information from authors about some types of materials, experimental systems and methods used in many studies. Here, indicate whether each material, system or method listed is relevant to your study. If you are not sure if a list item applies to your research, read the appropriate section before selecting a response.

Materials & experimental systems

n/a	Involved in the study	
☐	☒	Antibodies
☐	☒	Eukaryotic cell lines
☒	☐	Palaeontology and archaeology
☐	☒	Animals and other organisms
☒	☐	Clinical data
☒	☐	Dual use research of concern
☒	☐	Plants

Methods

n/a	Involved in the study	
☒	☐	ChIP-seq
☒	☐	Flow cytometry
☒	☐	MRI-based neuroimaging

Antibodies

Antibodies used	rabbit α-vinculin (Abcam ab129002, 1:2000), mouse α-tubulin (Abcam ab7291, 1:2500), rabbit α-IAV PB2 (GeneTex GTX125926, 1:2000), rabbit α-IAV PB1 (GeneTex GTX125923, 1:500), rabbit α-IAV PA (GeneTex GTX118991 1:500) mouse α-FLAG (Sigma-Aldrich F1804, 1:500), rabbit α-Gaussia luciferase (Invitrogen; PA1-181, 1:1000), sheep anti-rabbit IgG, HRP (Sigma-Aldrich AP510P, 1:20,000) and goat anti-mouse IgG, HRP (Bio-Rad STAR117P 1:1000) or goat anti-rabbit IgG IRdye800CW (Abcam ab216773, 1:20,000) and goat anti-mouse IgG IRdye680RD (Abcam ab216776, 1:20,000)
Validation	rabbit α-vinculin (Abcam ab129002) has been referenced in 206 published articles (https://www.abcam.com/products/primary-antibodies/vinculin-antibody-epr8185-ab129002.html?productWallTab=Abreviews).<br>mouse α-tubulin (Abcam ab7291) has been referenced in 887 published articles (https://www.abcam.com/products/primary-antibodies/alpha-tubulin-antibody-dm1a-loading-control-ab7291.html?productWallTab=Questions)<br>rabbit α-IAV PB2 (GeneTex GTX125926) has been referenced in 57 published articles (https://www.genetex.com/Product/Detail/Influenza-A-virus-PB2-protein-antibody/GTX125926#references)<br>rabbit α-IAV PB1 (GeneTex GTX125923) has been referenced in 49 published articles (https://www.genetex.com/Product/Detail/Influenza-A-virus-PB1-protein-antibody/GTX125923)<br>rabbit α-IAV PA (GeneTex GTX118991) has been referenced in 41 published articles (https://www.genetex.com/Product/Detail/Influenza-A-virus-PA-protein-antibody/GTX118991)<br>mouse α-FLAG (Sigma-Aldrich F1804) has been referenced in 8250 published articles (https://www.sigmaaldrich.com/GB/en/product/sigma/f1804).<br>rabbit α-Gaussia luciferase (Invitrogen; PA1-181) (validated in https://www.fishersci.co.uk/shop/products/anti-gaussia-luciferase-polyclonal-thermo-scientific/15507301)<br>sheep anti-rabbit IgG, HRP (Sigma-Aldrich AP510P) (validated in https://www.sigmaaldrich.com/GB/en/product/mm/ap510p)<br>goat anti-mouse IgG, HRP (Bio-Rad STAR117P) (validated in https://www.bio-rad-antibodies.com/polyclonal/mouse-igg-antibody-star117.html?f=purified#references)<br>goat anti-rabbit IgG IRdye800CW (Abcam ab216773) has been referenced in 66 published articles (https://www.abcam.com/products/secondary-antibodies/goat-rabbit-igg-hl-irdye-800cw-preadsorbed-ab216773.html)<br>goat anti-mouse IgG IRdye680RD (Abcam ab216776) has been referenced in 25 published articles (https://www.abcam.com/products/secondary-antibodies/goat-mouse-igg-hl-irdye-680rd-preadsorbed-ab216776.html)

Eukaryotic cell lines

Policy information about cell lines and Sex and Gender in Research

Cell line source(s)	MDCK and 293T cell lines were obtained from ATCC. eHAP cells were purchased from Horizon Discovery.
Authentication	These cell lines were not authenticated.
Mycoplasma contamination	These cell lines were not tested for mycoplasma.
Commonly misidentified lines (See ICLAC register)	No commonly misidentified cell lines were used in this study.

Animals and other research organisms

Policy information about studies involving animals; ARRIVE guidelines recommended for reporting animal research, and Sex and Gender in Research

Laboratory animals	Six to eight week old female BALB/c (Envigo RMS UK Ltd) mice.<br>20-24°C temperature<br>Humidity between 45-65%<br>12hour light-dark cycle
Wild animals	N/A
Reporting on sex	All mice were female.
Field-collected samples	N/A
Ethics oversight	All work was approved by the local genetic manipulation (GM) safety committee of Imperial College London, St Mary's Campus (centre number GM77), and the Health and Safety Executive of the United Kingdom.

Note that full information on the approval of the study protocol must also be provided in the manuscript.
